# Cure Kinetics of Epoxy Nanocomposites Affected by MWCNTs Functionalization: A Review

**DOI:** 10.1155/2013/703708

**Published:** 2013-11-19

**Authors:** Mohammad Reza Saeb, Ehsan Bakhshandeh, Hossein Ali Khonakdar, Edith Mäder, Christina Scheffler, Gert Heinrich

**Affiliations:** ^1^Department of Resin and Additives, Institute for Color Science and Technology, P.O. Box 16765-654, Tehran, Iran; ^2^Department of Polymer Processing, Iran Polymer and Petrochemical Institute, Tehran 14965-115, Iran; ^3^Leibniz-Institute of Polymer Research Dresden, 01069 Dresden, Germany; ^4^Technische Universität Dresden, Institut für Werkstoffwissenschaft, 01062 Dresden, Germany

## Abstract

The current paper provides an overview to emphasize the role of functionalization of multiwalled carbon nanotubes (MWCNTs) in manipulating cure kinetics of epoxy nanocomposites, which itself determines ultimate properties of the resulting compound. In this regard, the most commonly used functionalization schemes, that is, carboxylation and amidation, are thoroughly surveyed to highlight the role of functionalized nanotubes in controlling the rate of autocatalytic and vitrification kinetics. The current literature elucidates that the mechanism of curing in epoxy/MWCNTs nanocomposites remains almost unaffected by the functionalization of carbon nanotubes. On the other hand, early stage facilitation of autocatalytic reactions in the presence of MWCNTs bearing amine groups has been addressed by several researchers. When carboxylated nanotubes were used to modify MWCNTs, the rate of such reactions diminished as a consequence of heterogeneous dispersion within the epoxy matrix. At later stages of curing, however, the prolonged vitrification was seen to be dominant. Thus, the type of functional groups covalently located on the surface of MWCNTs directly affects the degree of polymer-nanotube interaction followed by enhancement of curing reaction. Our survey demonstrated that most widespread efforts ever made to represent multifarious surface-treated MWCNTs have not been directed towards preparation of epoxy nanocomposites, but they could result in property synergism.

## 1. Introduction

Since discovery and bulk synthesis of the new type of molecular carbon structures consisting of needle-like tubes in 1991, many research efforts have been devoted to explore the influence of incorporation of carbon nanotubes (CNTs) into different matrices [1]. The unique structure of CNT itself provides a rather wide range of extraordinary characteristics, for example, excellent electrical and thermal conductivities, low density, high aspect ratio, high surface area, and superior mechanical properties. The first paper on dealing with preparation of CNT/polymer nanocomposites in 1994 has encouraged many researchers to develop a new class of reinforced materials with synergistic properties via incorporation of CNTs in a variety of polymers [2]. Until now, both types of CNTs, that is, single-walled CNTs (SWCNTs) and multi-walled CNTs (MWCNTs), have been occasionally incorporated into different thermoplastics, elastomers, and thermoset polymers.

Studies have revealed that the rheological characteristics of thermoplastic nanocomposites, for example, PP/MWCNT [3], poly(methyl methacrylate) (PMMA)/SWCNT [4], high density polyethylene (HDPE)/MWCNT [5], polystyrene/MWCNT [6], and polycarbonate/MWCNT [7], are affected by the presence of carbon nanotube as well as increasing its content. The improvement of the mechanical, thermal, and optical properties has also been addressed in the literature. At low frequencies, the complex viscosity of thermoplastic matrices significantly increases with the addition of CNTs, whereas the loss modulus of nanocomposites undergoes a plateau, indicating the formation of percolated CNT networks that respond elastically over long time scales. The rheological percolation threshold of CNT/polymer nanocomposites obviously depends on temperature. On the other hand, the electrical conductivity in these systems is strongly governed by the state of dispersion, that is, nanotube-nanotube distance and alignment of CNTs in the matrix [3–6].

So many research efforts have also been dedicated to produce various thermoset composites containing CNTs. Epoxy resins have attracted much attention among thermoset family of materials owing to their excellent properties, for example, high modulus, low shrinkage in cure, and good chemical and corrosion resistance as well as acceptable adhesion characteristics. Besides, epoxies can be cured using a various ranges of chemicals with different types of curing conditions [8]. Some authors have addressed the cure behavior of epoxide thermoset composites filled with different types of nanosized fillers. Thermal studies on cure phenomenon of thermoset composites can be classified into two general categories: isothermal and nonisothermal kinetics. Domínquez et al. used different amounts of an acid catalyst based on p-toluenesulfonic acid dissolved in water (45% aqueous solution) to study the nonisothermal curing kinetics of polyfurfuryl alcohol bioresin [9]. Employing different isoconversional (nonisothermal) methods, that is, *Kissinger-Akahira-Sunone* (*KAS*), *Flynn-Wall-Ozawa* (*FWO*) and *Vyazovkin* (*VA*), they elucidated that the initial and final stage of curing, respectively, possesses an acceleration and deceleration period depending on the content of catalyst used. The limited movement of polymer chains and/or accumulation of reaction ingredients were the main reasons for the alteration of the cure mechanism. Several epoxide composites filled with CNTs have already been studied by different authors: bisphenol-A glycidol ether epoxy/2-ethyl-4-methylimidazole (DGEBA/EMI-2,4) system containing pristine and amine functionalized MWCNTs by Yang et al. [10, 11]; liquid-crystalline epoxy system of 4,4′-dihydroxy-a-methyl-stilbene/sulfanilamide containing carbon nanotubes and carbon black conventional filler by Bae et al. [12]; DGEBA/EMI-2,4 system containing purified MWCNTs by Zhou et al. [13]; DGEBA/4,4′-ethylenedianiline (DDM) epoxide systems containing amine functionalized MWCNTs by Prolongo et al. [14]; DGEBA/diethyltoluenediamine systems with carboxyl and fluorine modified MWCNTs by Abdalla et al. [15]; DGEBA/DDM composites comprising amine functionalized MWCNTs by Choi et al. [16]; DGEBA/1,3-phenylenediamine epoxy-amine CNT-free system by Sbirrazzuoli et al. [17]; DGEBA/diethylenetriamine (DETA) containing pristine SWCNTs by Puglia et al. [18]; tetraglycidyl-4,4′-diaminodiphenylmethane (TGDDM)/4,4′-diaminodiphenylsulfone (DDS) system comprising MWCNTs by Xie et al. [19]; DGEBA/Novolac as an epoxy/phenolic system containing phenol anchored multi-walled carbon nanotube by Choi et al. [20]; and MWCNTs/DGEBA/EMI-2,4 nanocomposites containing carboxylic functionalized MWCNTs by Zhou et al. [21].

Similar to the case of thermoplastic composites, CNT/epoxy thermoset systems have been the subject of several studies to investigate characteristics other than curing, for example, the rheological and mechanical properties. For many different reasons, which will be discussed in the next sections, surface treatment or functionalization of CNTs greatly enhances the performance of prepared nanocomposites. Hadjiev et al. used Raman spectroscopy to measure the magnitude of residual stresses in diglycidyl ether of bisphenol-F (DGEBF) epoxide systems cured with diethyltoluenediamine (DETDA) [22]. They prepared different types of CNT/epoxy composite samples employing pristine and functionalized. Kaffashi et al. discussed the extent of improvement in rheological characteristics in DGEBA/metaphenylene diamine (M-PDM) systems containing covalently modified and remodified MWCNTs [23]. Primarily, the MWCNTs were surface modified with a mixture of sulfonic and nitric concentrated acids and then diluted with distilled water. To produce remodified MWCNTS, in-situ esterification of carboxylated MWCNTs was performed. There were significant shifts in loss and storage modulus in specimens prepared by modified and remodified MWCNTs. Depending on the conducted strategy, various features have been considered while performing a specified test on the samples, as the work done by Puglia et al. [24]. Differently from the way used by Hadjiev et al., Puglia and coworkers discussed the impact of SWNTs on the cure reaction of DGEBA/DETA composites by means of thermal analysis and Raman spectroscopy. They revealed that SWNTs act as a strong catalyst and reduce the temperature assigned for the exothermic reaction peak. Carboxylated and fluorinated nanotubes were used by Abdalla et al. to synthesize nanocomposites by dispersing them separately in DGEBA/diethyltoluenediamine (DETDA) thermoset system [25]. Attention has been made by the authors to investigate the role of interfacial chemistry in molecular mobility and morphology of produced nanocomposites. Kim et al. reported the mechanical and rheological properties of diglycidyl ether of bisphenol-A epoxide systems (YD 128) cured by a modified aromatic amine hardener (TH 432) [26]. Based on amine treatment and plasma oxidation, they attempted to improve the interfacial adhesion as well as the performance of dispersion of multi-walled nanotubes in the epoxy matrix. The degree of performance of CNT/epoxy systems greatly depends upon the state of dispersion of nanotubes in the epoxy matrix. Song and Youn demonstrated that the rheological, mechanical, electrical, and thermal properties of YD 128/TH 432 epoxy are governed by the state of dispersion of MWCNTs, whether a solvent is used or not [27]. In epoxy-based systems and/or their reinforced composites, Doan et al. have made a lot of efforts to interpret the alteration of some vital properties, mostly adhesion and mechanical characteristics, on account of interphase situation. From a practical point of view, the following can be considered as the most important works performed by collaboration or under supervision of Mäder: incorporation of MWCNT and glass fiber into a DGEBA-based epoxy resin to the formation of semiconductive MWCNT-glass fibers and in turn multifunctional fiber/polymer interphases [28]; evaluation of healing efficiency and tensile strength of glass fibers with MWCNT/DGEBA/m-phenylenediamine (m-PDA) nanocomposite coating affected by the type and content of carbon nanotubes used which conversely influenced the dispersion state [29]; evaluation of the adhesion and interlaminar shear strengths [30, 31] and surface roughness in different epoxy composites containing poly(p-phenylene-2,6-benzobisoxazole) (PBO) fibers via surface modification of PBO [32]; measuring the static (constrained molecular mobility of polymer chains) and dynamic (interfacial adhesion and the tendency of fatigue resistance) properties of single and multifiber/DGEBA-based epoxy composites modified by sizing [33]; modification of epoxy-based composites by polysulfone to improve the interfacial and mechanical properties in its glass fiber composites [34]; tracking the topography, fractography, and interphases in carbon fiber/epoxy composites [35]; and bond strength measurement between glass fibers and epoxy resin at elevated temperatures using the pull-out and push-out techniques [36, 37].

Despite the fact that using CNTs may itself affect the electrical, mechanical, or rheological properties, it has been proved that the main drawback of incorporation of CNTs into the polymers is the formation of bundles or entanglements consisting of hundreds of individual particles by van der Waals force, as a consequence of heterogeneous distribution within the host material. In another words, CNTs are very potent to form entangled structures because of small diameter in nanometer scale and high aspect ratio (normally > 1000). For epoxide nanocomposites, the reagglomeration of CNTs after curing reaction has also been reported. Thus, ultimate performance of epoxy and/or other thermoset resins containing nontreated CNTs intensely depends upon the state of dispersion. Several techniques have been proposed by the researchers for overcoming this problem. Hilding et al. indicated that during grinding of MWCNTs much of the mechanical energy goes into complete breakup of tubes and new defects will be continuously generated on the tube surface [38]. They comprehensively gathered the available data to give emphasize to the effects of milling, ultra sonication, high shear flow, elongational flow, functionalization, and surfactant and dispersant systems on morphology of carbon nanotubes and their interactions in the fluid phase. As a model system, MWCNTs have been considered for experimental work owing to their accessibility in engineering-scale quantities and dispersed reproducibly in a variety of solvents and polymers. To deepen the understanding of effective dispersion, Ma et al. reviewed the state of dispersion of CNTs throughout the different polymers [39]. They compared the total surface area of well-known conventional micro- and nanoscale fillers and served a 3D illustration to visualize the nature of dispersion problem for CNTs. Accordingly, dispersion of CNTs in organic solvents accompanied by sonication results in individually dispersed nanotubes in epoxide systems. It was also concluded that the time of sonication as well as the solubility parameter of the solvent greatly affects the dispersion/debundling process of untreated CNTs. Matarredona et al. employed an anionic surfactant, namely, sodium dodecylbenzenesulfonate (NaDDBS), to improve the suspendability of SWCNTs in aqueous solutions [40]. The degree of interaction between nanotubes and the used surfactant was examined by varying the pretreatment method, that is, acidic or basic purification. After chemical functionalization of nanotubes, however, it was found that the structure of surfactant-stabilized SWCNTs was governed by the hydrophobic forces between the surfactant tail and the nanotube. In fact, each nanotube was seemingly covered by a monolayer of surfactant molecules in which the heads formed a compact outer surface while the tails remained in contact with the nanotube walls. The effects of sonication time and concentration of NaDDBS on suspendability of SWNTs have also been reported. Pizzuttoa et al. examined several parameters, for example, CNT content, sonication time, sonication power, the use of solvent, the amount of surfactant, and the influence of degassing on the state of dispersion in SWCNT-appended DGEBA-based systems [41]. Among two types of SWCNTs used, that is, the pristine and carboxylated SWCNTs, the latter resulted in lower time and amplitude of sonication leading to higher mechanical properties, namely, Young's modulus, tensile strength, and breaking elongation. Likewise, Gkikas et al. considered dispersion state of MWCNT/epoxy nanocomposites by taking the CNT content, the sonication time and the total sonication energy as the inputs [42]. They found that the sonication energy, as the most influential parameter, determines the toughness properties. Besides, the best condition leading to augmentation of glass-rubber transition temperature (*T*_*g*_) and storage modulus was achieved after 2 h of sonication and 50% sonication amplitude. Chapartegui et al. speculated that the shear thinning behavior observed in their amine cured MWCNT/GGEBA system is expectable as a result of relatively low molecular weight of the epoxy used (700 g/mol corresponding to a single repeating unit) [43]. When choosing such a system, a physical network comes into being in which the carbon nanotubes are able to get in touch with each other rather than combined CNT/polymer network interactions. The shear-induced rheological test might have destructive effect on the network; nevertheless, the rapid recovery of the MWCNT network in the prepolymer matrix was responsible for the lower electrical threshold than that of rheological one.

Another scheme to resolve poor dispersion of CNTs in epoxy is chemical functionalization of CNTs. Until now, a variety of strategies have been developed for functionalization of CNTs [44]. Moreover, the effect of treated nanotubes on the state of dispersion as well as ultimate properties of CNT/polymer nanocomposites has deeply been concerned [39]. A detailed literature survey towards the effect of surface modification on dispersion state of carbon nanotubes in solvents and polymers have been summarized by Kim et al. [45]. To properly determine the relationship between surface characteristics and dispersibility of CNTs, some terminologies has been employed, for example, the degree of surface modification, degree of substitution, and degree of dispersion. The comparative approach used by the authors on gathered information from the literature, that is, tabulated data and illustrative perspectives, fulfills a need for functionalization to achieve appropriate dispersion of CNTs in aqueous solutions. In general, both covalent and noncovalent surface modifications have been examined. To draw a convincing conclusion on quantitative studies, the *Flory-Huggins* interaction parameter was considered as a measure of solubility parameter for CNTs, corresponding to dispersibility in the surrounding media. Park et al. compared the thermal conductivity of GDEBA-based composites containing short and long MWCNTs [46]. Employing the long-MWCNTs resulted in higher electrical and thermal conductivities, especially when the degree of alignment was increased by mechanical stretching. Albeit the epoxidation of CNT improved the mechanical properties of specimens, CNT walls were damaged by peroxide acid treatment and decreased the electrical conductivity. Apparently the SWCNTs functionalized with epoxide grafting dissolve in organic solvents, for example, dimethyl formamide, chloroform, and methylene chloride with relative ease [47]. Other papers on the effect of CNT functionalization are also accessible in the literature: the improvement of the dispersion state and thermomechanical properties of DGEBA systems using covalently and noncovalently functionalized MWCNTs by Damian et al. [48]; elaborating on the use of chemically functionalized MWCNTs with aromatic amines to investigate the cure behavior, mechanical properties, thermal stability, and fracture morphology of DGEBA-based composites by Ghorabi et al. [49]; improving the mechanical properties of epoxy composites using MWCNTs functionalized by a novel plasma treatment by Chen et al. [50]; investigation of the effects of various types of functionalized MWCNTs (carboxylated (MWCNT-COOH) and directly fluorinated (MWCNT-F)) on thermomechanical and morphological properties of epoxy based nanocomposite systems by Theodore et al. [51].

Although a large number of publications have been devoted to functionalization of carbon nanotubes, there have only been a few papers on dealing with cure kinetics of epoxy-based systems influenced by the functionalization of CNTs. Also, certain discrepancies in the kinetic data might sometimes happen owing to various reasons, for example, inappropriate dispersion of nanotubes, functionality of the used epoxy, the type of curing agent, cure condition, and the type of CNT, which makes the interpretation of cure mechanism in epoxy nanocomposites quite difficult.

Allaoui and El Bounia reviewed and analyzed the effect of untreated SWCNTs and MWCNTs on the cure behavior of epoxy resins with emphasis on alteration of *T*_*g*_ [52]. They compared the difference between *T*_*g*_ of nanocomposites and that of the neat resin and made sufficient effort to find a trend through the data reported in the literature. Their interpretations based upon the CNT type (SWCNT or MWCNT), dispersion method (grinding, dissolution, using surfactants, sonication or combinatorial methods), percent by weight of CNT, and the aspect ratio of the used nanotube, indicate that incorporation of SWCNTs in epoxy decreases the *T*_*g*_ due to rather high bundling tendency, while the use of MWCNTs often suggests an increased or unchanged *T*_*g*_. The acceleration effect of both types of CNTs in the early stage of curing of epoxide system was also reported. The influence of unmodified nanotubes on cure kinetics will be reviewed in the next sections. Irrespective of the type of curing agent and thermal history, it was generally agreed that the cure reaction in epoxy/CNT nanocomposites is very sensitive to the surface treatment.

This paper attempts to highlight the influence of functionalization of MWCNTs on the cure behavior of epoxy composites. The autocatalytic, noncatalytic, and vitrification mechanisms have also been considered through a literature review on isothermal and nonisothermal curing schemes by calorimetry.

## 2. The Chemistry of Curing in Epoxy Composites

**Scheme 1:**
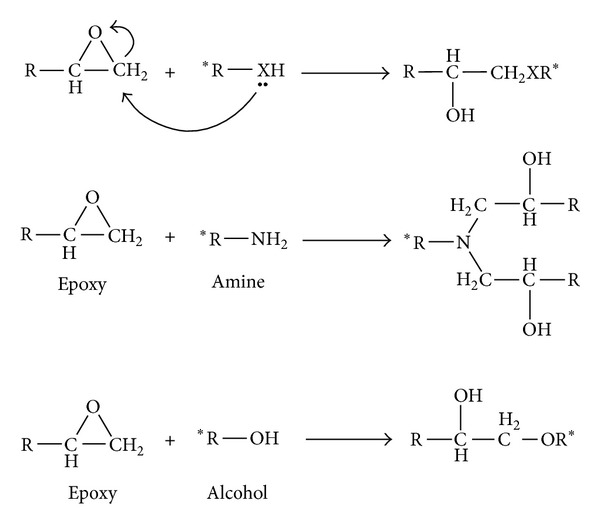
Oxirane ring opening via the nucleophilic addition reaction in an epoxide system.

Epoxy resins belong to a class of thermosetting materials containing two or more oxirane rings or epoxy groups in their molecular structure. The performance of an epoxy-based composite significantly depends on its curing circumstance [54, 55]. Studies on curing behavior of such systems demonstrated that the epoxide molecules contributed to the curing reaction and react with themselves to form a crosslinked network and/or with other reactive molecules whether or not a catalyst is used [39]. Depending on the type of curing agent, for example, amines, thiols, alcohols, and anhydrides, as well as curing condition, that is, isothermal and nonisothermal, it is often possible to predict the final application of the cured resin. As seen in Scheme 1, the curing reaction takes place with oxirane ring opening via the nucleophilic addition reaction.

**Scheme 2:**
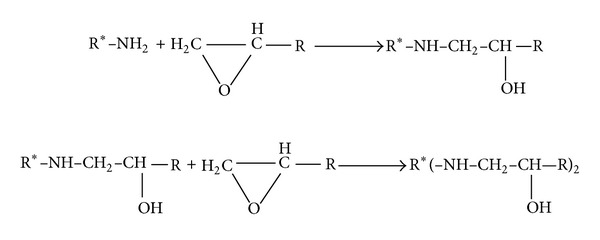
Mechanism of curing epoxy resins with amine hardeners.

Curing of epoxide groups with amine hardeners revealed that the primary amine hydrogen reacts with the epoxy resin and subsequently the secondary amine hydrogen comes into exist which can react with the other epoxy ring.  Scheme 2 illustrates the mechanism of curing of epoxy resins with amine hardeners [56].

**Scheme 3:**
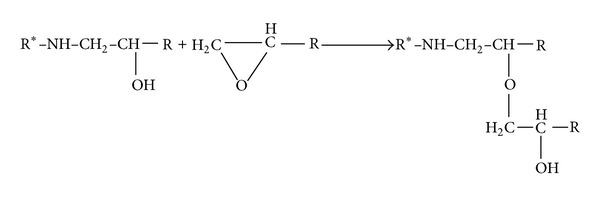
Illustrating the mechanism of etherification reaction.

At the same time, the hydroxyl groups generated by the reaction between the secondary amine hydrogen and epoxy result in formation of ether links. This reaction, namely, etherification, competes with the amine-epoxy cure reaction, as shown in Scheme 3 [8, 56].

In case of low reactivity of the amine group or when there is an excess epoxy ring in the backbone, this competition causes a fluctuation in the cuing rate and, therefore, makes the interpretation of cure mechanism quite difficult.

**Scheme 4:**
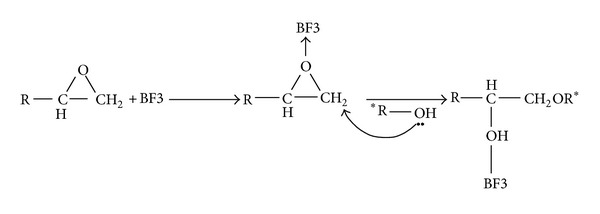
Mechanism of Lewis acid-catalyzed curing of epoxy by alcohol.

It has been generally agreed that the extent of etherification significantly depends on cure temperature and epoxy/amine system, for example, the stoichiometric ratio of resin to hardener in the mixture and the functionality of epoxy resin. Also, the secondary alcohols continuously formed during cure reaction may accelerate the amine-epoxy reactions. Thus, two mechanisms compete against each other for curing in the system. The first one is an autocatalytic reaction which occurs because of hydroxyl groups initially existed in the epoxy prepolymer or those formed during the reaction. The autocatalytic mechanism involves a ternary transition complex (Scheme 4).

**Scheme 5:**
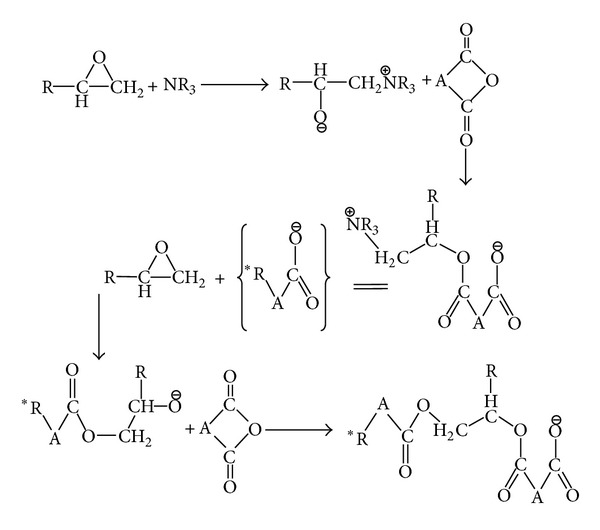
Mechanism of curing epoxy resins with anhydride hardeners.

At elevated temperatures, the catalytic mechanism almost vanishes due to the difficulty of forming such a ternary complex. The second mechanism proposed for the cure of epoxide systems, which is a second-order noncatalytic reaction in nature, takes place over the entire range of temperature [8]. As the fractional extent of conversion increases, the *T*_*g*_ of the network goes higher and becomes identical to the cure temperature. This transition state is called vitrification and depends upon cure temperature and reaction kinetics. In this situation, the occurrence of etherification reaction is highly probable [8, 19, 56]. Acid anhydrides have extensively been employed as the second commonly used candidates, after amine hardeners, for the curing of epoxy monomers [57]. The cured epoxies by use of anhydride agents are suitable for especial applications, for example, coatings and electronic devices. The main difference between the amine-epoxy and the anhydride-epoxy reactions is that the latter undergoes a chain-wise polymerization, on the contrary with the former which passes through a stepwise mechanism. Therefore, the anhydride-epoxy reactions involve an initiation by Lewis bases, as well as propagation and termination or chain transfer steps [57]. Some of the postulated reactions are shown in Scheme 5.

As in Scheme 5, the initiation step proceeds with ring opening of an epoxy monomer by aim of a tertiary amine. In the next step, the active anion reacts with an anhydride group very quickly to form a carboxylate anion as the active site. This esterified constituent contributes to the cure reaction as an initiator of the chain-wise polymerization. The number of active sites affects the content of Lewis base initiator and subsequently makes this constituent potent to react with the other epoxy group which again attacks the cyclic anhydride. As all the mentioned mechanisms are temperature dependent, differential scanning calorimetry (DSC) leads to convincing conclusions on the cure kinetics of epoxy composites. The model-fitting and model-free kinetic approaches have occasionally been employed by researchers to assess the cure kinetics of epoxy nanocomposites. The model-fitting method often leads to some inaccuracies or kinetic compensation effect, whereas the model-free or isoconversional method is not sensitive to cure kinetic [9, 17, 58–64].

## 3. Cure Kinetics of Epoxy/MWCNTs Nanocomposites

**Figure 1:**
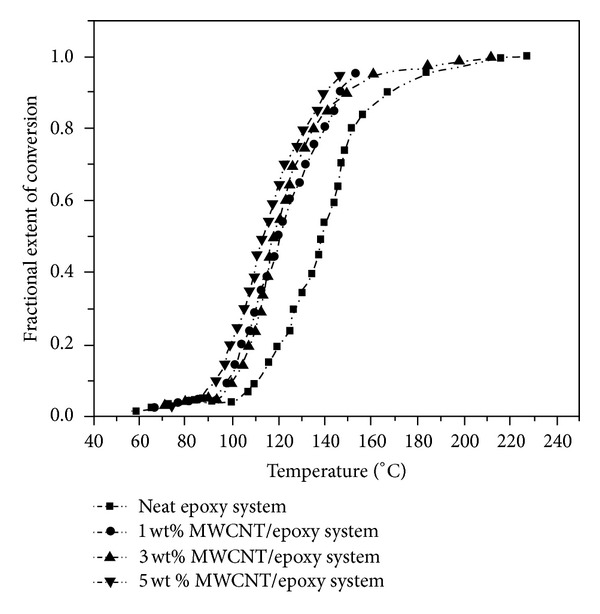
Conversion as a function of temperature at heating rate of 20°C/min for the neat epoxy and MWCNTs-filled epoxy systems. After [13].

Zhou et al. demonstrated that the use of MWCNTs facilitates crosslinking at the initial curing stage of bisphenol-A glycidol ether epoxy/2-ethyl-4-methylimidazole (DGEBA/EMI-2,4) system by lowering the peak temperature and the heat of reaction, in turn, prevents the occurrence of vitrification by lowering the *T*_*g*_ compared to neat epoxy resin [13]. At higher contents of MWCNTs, however, the overall degree of cure declines due to the reduction of epoxy concentration and probable agglomeration. Dynamic DSC determination often leads to sigmoidal form of conversion-temperature curves being observed indicating an autocatalytic kinetic in almost all studied systems. As illustrated in Figure 1, the addition of MWCNTs does not change the autocatalytic cure reaction mechanism of DGEBA/EMI-2,4 system. Also, a small variation in the amplitude of heat of cure implies that the etherification reaction is dominant in the cure process.

In case of isothermal curing of epoxide systems, several authors employed *Kamal* equation to investigate the extent of autocatalytic reaction [10, 12, 14–19, 24], as follows:

(1) $$\begin{array}{c} \frac{d\alpha }{dt}=\left(k_{1}+k_{2}\alpha^{m}\right){\left(1-\alpha \right)}^{n}, \end{array}$$

where *k*_1_ and *k*_2_ are the autocatalytic and noncatalytic rate constants, *m* and *n* are kinetic exponents, and *α* is the fractional conversion of cure reaction, respectively.

Xie et al. studied the cure kinetics of tetraglycidyl-4-4′-diaminodiphenylmethane/4,4′-diaminodiphenylsulfone (TGDDM/DDS) systems containing MWCNTs through isothermal calorimetry [19].

**Figure 2:**
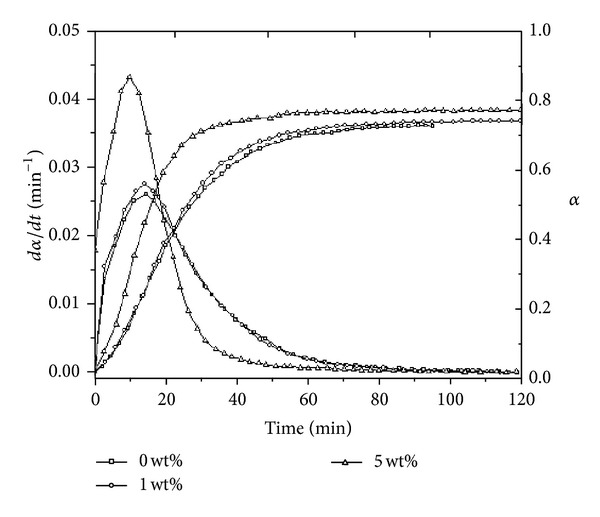
The reaction rate and extent of reaction of TGDDM/DDS epoxy and its nanocomposites as a function of time at 180°C. After [19].

The reduction of the time to the maximum rate with increasing MWCNTs concentration typically proved the early stage autocatalytic reaction. In addition, the experimental results agreed with kinetic model of *Kamal*. They plotted the reaction rate and the extent of reaction of TGDDM/DDS and its nanocomposites as a function of time at different temperatures. The one displayed at 180°C is given in Figure 2, for instance.

**Figure 3:**
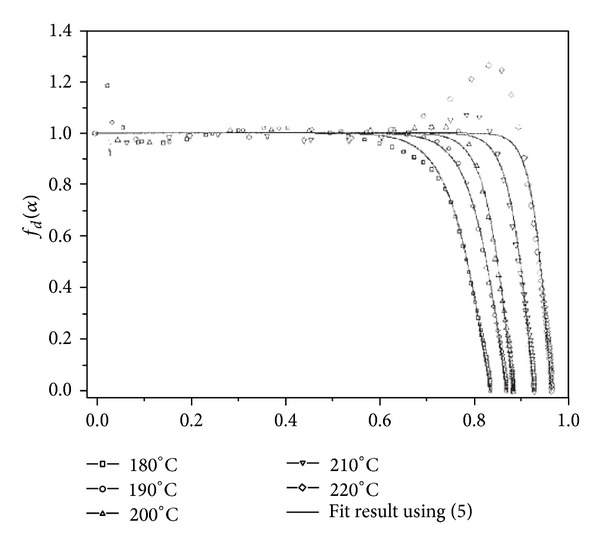
Curve of diffusion control function, *f*_*d*_(*α*), against extent of reaction for 1 wt% MWCNTs/epoxy nanocomposites: the effect of curing temperature. After [19].

The authors modified *Kamal* equation multiplying the right side of (1) by a diffusion control function to express the contribution of diffusion to cure reaction.  Figure 3 illustrates the effect of curing temperature on diffusion control function, namely, *f*_*d*_(*α*).

If the reaction is chemically controlled, *f*_*d*_(*α*) is unity, whereas in the case of full diffusion control the reaction is practically interrupted and the diffusion control is zero. As seen in this figure, at curing temperatures beyond 200°C, when *α* is greater than 0.7, the reaction rate significantly increases up to the value predicted by *Kamal* Model until drop-off due to diffusion. This deviation in *f*_*d*_(*α*) demonstrates that curing is diffusion controlled because of vitrification.

It was also mentioned that this unexpected increase in the value of *f*_*d*_(*α*), which is observed in case of neat epoxy and 1 wt% of MWCNTs/epoxy systems, stems mainly from the etherification. Thus, it can be concluded that the saturation of catalyzing action is often probable in highly filled epoxy nanocomposites with unmodified MWCNTs. Similar trend was observed in DGEBA/diethylenetriamine (DETA) nanocomposites containing SWCNTs [18, 25].

## 4. The Influence of Functionalization of MWCNTs on Cure Kinetics

**Figure 4:**
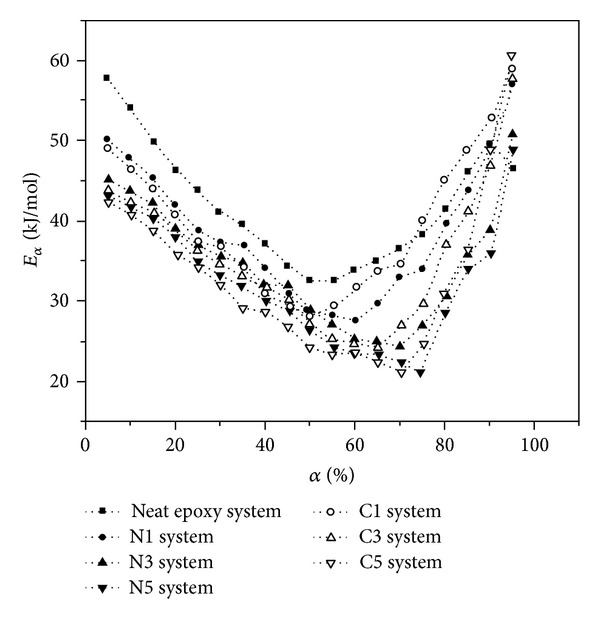
Plots of activation energy against *α* of neat epoxy, untreated systems containing 1, 3, and 5 wt% of MWCNTs (N1, N3, N5), and functionalized systems containing 1, 3, and 5 wt% of COOH-MWCNTs (C1, C3, C5). Dotted lines are given for showing the tendency. After [21].

The CNTs bearing active groups can significantly change the surface characteristics of these constituents. These functional nanotubes can react with different materials such as resins or act as a catalyst, thus enhancing the interfacial bond between the matrix and the CNTs dictates the application of prepared polymer nanocomposites [39, 44]. Cure kinetics of an epoxide system is highly dependent on the nanocomposite constituents. In general, the reactivity of amine hardeners increases with their nucleophilic nature; therefore, employing an appropriate catalyst as well as a proper curing temperature leads to highly crosslinked networks [8]. On the other hand, the functionalization of CNTs increases their surface roughness. Such a modification can be performed physically or chemically. One of the major drawbacks of physical treatment is the lack of appropriate interfacial adhesion as a consequence of weakness of the van der Waals forces. In case of chemical modification; however, the interaction between nanotubes and the matrix is responsible for the strong adhesion due to formation of covalent bonds at the interface. A variety of techniques and also functional groups have been employed to give access to functionalization of CNTs [38–44]. Among different techniques, the carboxylation, halogenation, and amidation are the most commonly used methods for enhancing structural properties of CNT/epoxide composites. In general, after functionalization of nanotubes the polar groups located on the surface of filler act as curing agents and accelerate the cure reaction of epoxy. To our knowledge, functionalized MWCTNs bearing reactive groups, for example, –COOH and –NH_2_, have been considered to evaluate cure criterion. The presence of carboxylic and other oxygen-bearing groups at the surface of nanotubes and at defect sites promotes nanofiller reactivity. However, mainly due to the large aspect ratio of CNTs, the occurrence of sidewall functionalization is also probable [44]. Bae et al. modified two kinds of CNTs with carboxylic monomers towards liquid-crystalline epoxy and stated that the increase of heat of cure and decrease of activation energy justifies higher nucleophilicity after surface treatment [12]. In other words, polar interaction between carboxylic groups and epoxide facilitates the breakage of epoxide rings and promotes the homogeneous distribution of nanofillers. The effect of polar-polar interactions on the activation energy in such systems is more salient compared to steric hindrance. Furthermore, the isothermal kinetic parameters evaluated from *Kamal* equation provide support for the remarkable effect of oxidation in the early stage of cure reaction. Zhou et al. indicated that –COOH functionalization of MWCNTs does not change the autocatalytic cure mechanism of DGEBA/EMI-2,4/MWCNTs nanocomposites [13, 21]. At the early stages of curing, both types of MWCNTs, that is, the pristine and COOH-functionalized represent catalytic effect on the curing reaction. This influence is more pronounced in case of modified filler because the –COOH groups can react with epoxide hydroxyl group to form C–O–C ether bonds. At the later stages, however, the unmodified MWCNTs delay vitrification more than carboxylated fillers. The authors reasoned that –COOH groups improve the compatibility between the MWCNTs and epoxy matrix leading to lesser free volume and hindrance effect on vitrification phenomenon.  Figure 4 demonstrates that inflexion of activation energy curves at fractional conversions greater than 0.7 increases less with increasing COOH-MWCNTs content compared to nontreated nanotube, as a consequence of constrained segmental motion in this situation [21].

**Scheme 6:**
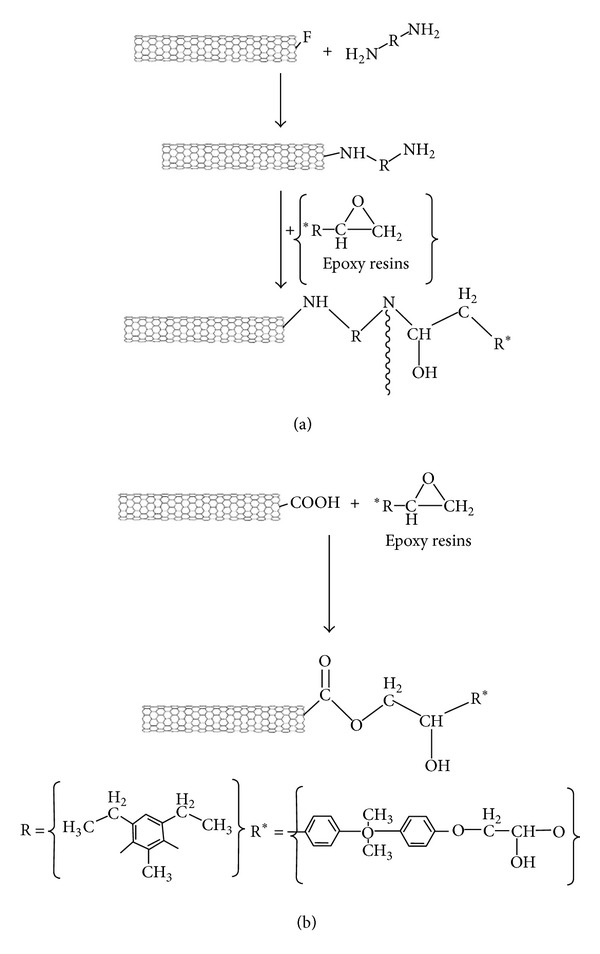
Schematic representation of the reaction between functionalized MWCNTs with DGEBA resin: (a) fluorinated CNT; (b) carboxylated CNT.

Abdalla et al. investigated the rheological and cure characteristics of DGEBA/EPIKURE systems comprising carboxylated and fluorinated MWCNTs [15, 25]. As seen in Scheme 6, the cure mechanism of the neat epoxy and fluorinated system is very similar, wherein the heat of cures is measured to be 47.5 and 47.7 kJ/mol, respectively.

**Figure 5:**
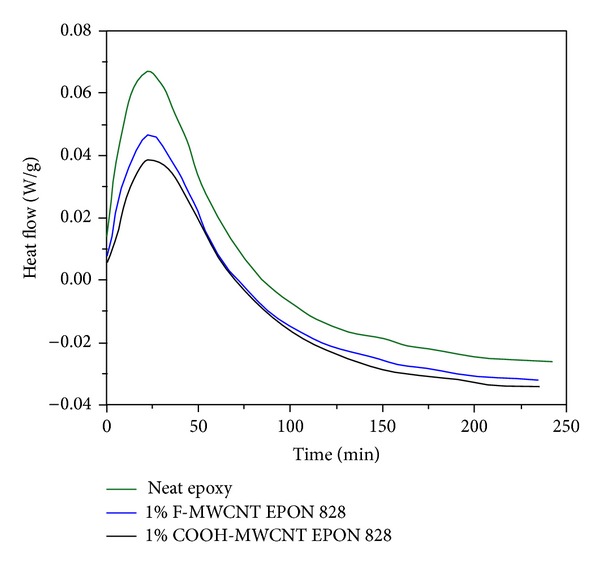
Isothermal DSC thermograms of neat EPON828, 1 wt% F-MWCNTs/epoxy and COOH-MWCNT/epoxy, nanocomposites at 120°C. After [15].

Contrarily, the higher heat of reaction in case of carboxylated nanotubes (61.7 kJ/mol) is indicative of a completely different cure mechanism. Nanotubes bearing carboxylic groups ease the ring opening and substantially generate an ester bond and an alcohol group, whereas the fluorinated CNTs essentially act as an amine curing agent. The rate constants determined by *Kamal* model revealed that in the fluorinated system CNTs are dispersed appropriately demonstrating quite a high surface area; in turn, the presence of aggregates in the carboxylated system causes insufficient catalytic effect (Figure 5).

At higher curing temperatures, for example, 140°C, reaction rates are almost independent of the surface modification. Also, samples containing fluorinated MWCNTs exhibit the highest *T*_*g*_ among all nanocomposites corroborating existence of uniformly dispersed nanotubes. The value of crosslink density calculated using the plateau modulus are almost the same, except for 1 wt% samples, where the fluorinated samples exhibit higher values. Significant heat capacity at higher temperatures in case of carboxylated nanocomposites is another evidence for constrained mobility of reacting species due to inhomogeneous dispersion. The authors speculated that the density of functional groups on the surface of CNTs alters by the type of modifier, which is responsible for this observation. The results provided support for the fact that further treatments on carboxylated MWCNTs, for example, in situ esterification with oligomeric unsaturated hydroxyl-terminated polyesters, intensely affect the rheological and fracture properties [23, 66].

**Scheme 7:**
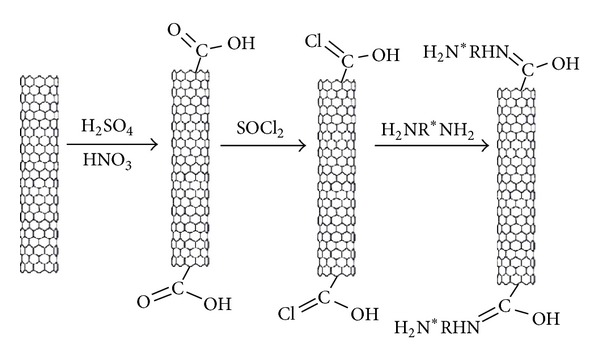
The procedures used for functionalization of MWCNTs by Shen et al.

The effect of amine functionalization on MWCNTs has also been studied by researchers. Shen et al. used the procedure illustrated in Scheme 7 to prepare modified MWCNTs with amide groups from carboxylated nanotubes [53, 65].

**Figure 6:**
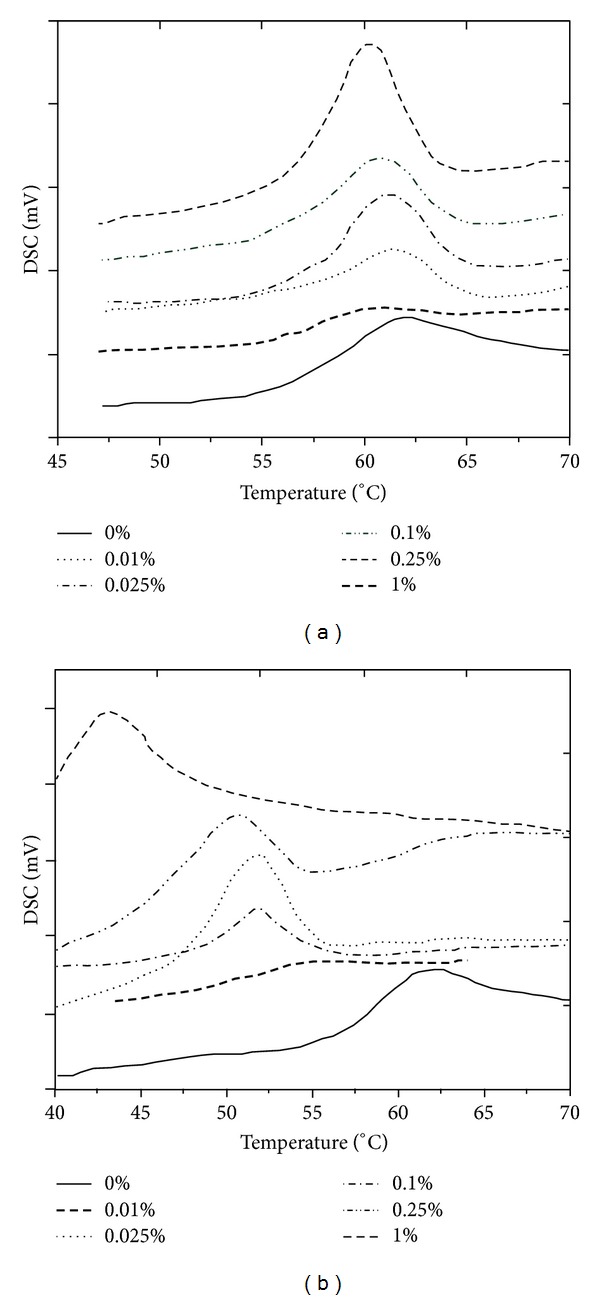
Dynamic DSC thermograms of neat epoxy and its raw MWCNTs nanocomposites (a); neat epoxy and its amino-functionalized MWCNTs nanocomposites (b). After [53].

They found that the initial decomposition temperature of nanocomposites increases about 30°C by addition of 0.25 wt% amino-functionalized MWCNTs corroborating strong interphase between epoxy and modified nanotubes. When the amount of nanotubes exceeds 1 wt%, the decomposition temperature slightly decreases. According to Figure 6, depression of the *T*_*g*_ of about 20°C observed when 1 wt% of amino-functionalized MWCNTs incorporated into composite.

**Figure 7:**
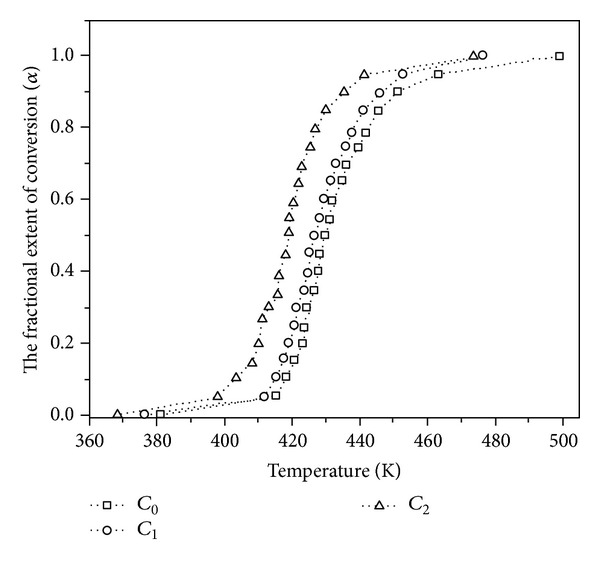
DSC thermograms at 20°C/min of neat epoxy (*C*_0_), 0.5 wt% as-received MWCNTs/epoxy nanocomposites (*C*_1_), and 0.5 wt% amine-modified MWCNTs/epoxy nanocomposites (*C*_2_). Dotted lines are given for showing the tendency. After [10].

This can be attributed to enhanced interfacial adhesion with epoxy. This research group also discussed for reinforcement mechanism of different amino-functionalized MWCNTs in epoxy resin. They found that the *T*_*g*_ of nanocomposites is obviously governed by the amidation because the amide groups participate in curing reaction as a hardener. Yang and collaborators compared cure behavior of EPON828/EMI-2,4 composites with those filled with pristine and amine-modified MWCNTs [9, 10]. For all prepared samples only one peak appeared throughout DSC thermograms, irrespective of the heating rate. Thus, etherification reaction dominantly occurred. As in Figure 7, addition of untreated or functionalized MWCNTs does not change the autocatalytic cure kinetics of epoxy.

**Figure 8:**
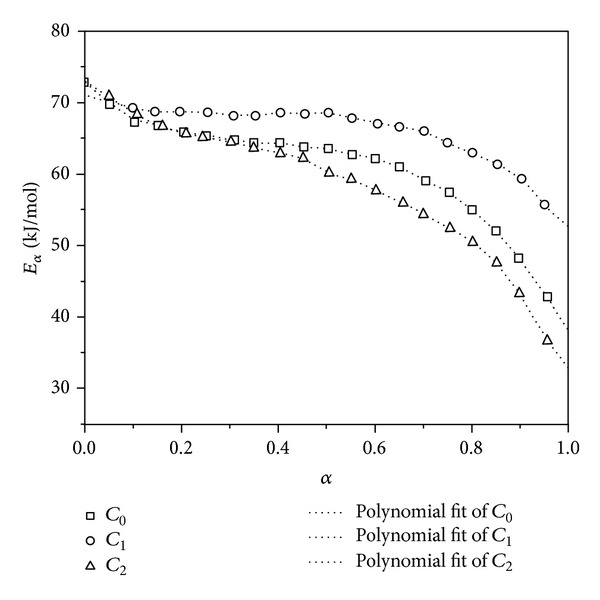
Plots of activation energy of neat epoxy (*C*_0_), 0.5 wt% as-received MWCNTs/epoxy nanocomposites (*C*_1_), and 0.5 wt% amine-modified MWCNTs/epoxy nanocomposites (*C*_2_) against degree of cure obtained by isoconversional kinetic model. After [10].

As-received nanotubes (C_1_) show retarding effect on the cure reaction of epoxy resin (C_0_) due to their hindrance, whereas the amino-functionalized MWCNTs (C_2_) accelerate the curing reaction as a secondary curing agent and facilitate the primary amine-epoxide reaction (Scheme 1). Furthermore, the alteration of activation energy as a function of extent of reaction demonstrates the accelerating effect of amine functionalized nanotubes on cure reaction, in particular at later stages of curing (Figure 8).

**Figure 9:**
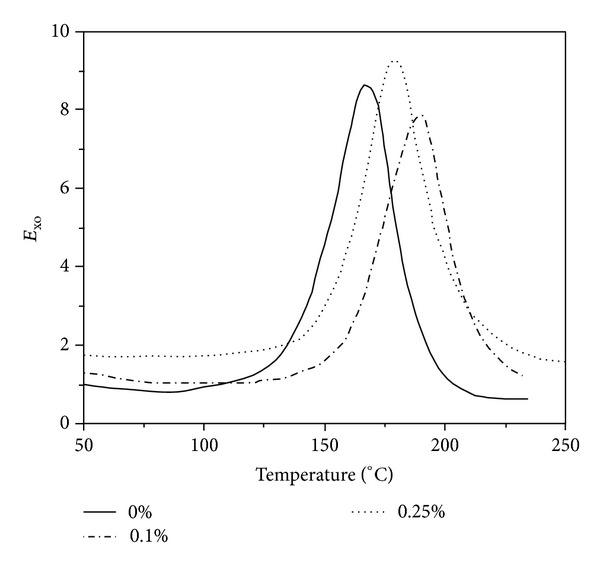
Dynamic DCS thermograms of stoichiometric mixtures (*r* = 1) with different amino-functionalized CNT contents. After [14].

The autocatalytic mechanism of curing is also examined by phenomenological model of *Kamal* and showed good agreement. The authors also reported that increasing the concentration of amino nanotubes up to 3 wt% results in an increase in the degree of vitrification. In this situation, reaction is likely to be diffusion controlled at lower heating rates and larger conversions because of significant steric hindrance. It means that accumulation of amine groups on the surface of MWCNTs intensifies the catalyzing effect during the epoxy cure reaction. Employing isothermal and nonisothermal calorimetric studies, a similar trend is observed in DGEBA/DDM composites containing 3 parts by weight of as-received and amine-treated MWCNTs based on 100 parts by weight of epoxy resin [16]. It can be concluded that the critical concentration in the functionalized epoxy nanocomposites seems to be around 1 wt%, as that of untreated/epoxy systems. Prolongo et al. prepared various DGEBA/DDM mixtures containing different amine functionalized MWCNTs (0, 0.1, 0.25 wt%) and changed the ratio of amine to epoxy, namely, *r*, as a measure of the amine hydrogen equivalents per oxirane rings [14]. Irrespective of the stoichiometry, only one exothermic peak appeared throughout dynamic DSC thermograms corresponding to amine/epoxy curing reaction.  Figure 9 displays dynamic DCS thermograms of stoichiometric mixtures (*r* = 1) with different amino-functionalized CNT contents.

In case of stoichiometric mixtures (*r* = 1), the heat of cure is lower than that of theoretical value for epoxy/amine reaction enthalpy (close to 95–100 kJ/ee) which suggests that the epoxy network is not completely crosslinked. This unexpected deviation is more pronounced for the mixture reinforced with 0.1 wt% nanotube. As the cure reaction promoted via step polymerization, the retardation effect could not be associated with diffusion controlled mechanism but with adsorption of monomers into the nanotubes which disrupts the reaction stoichiometry. When the concentration of amino nanotube increased, the imbalance in stoichiometry is supposed to be compensated; however, formation of aggregates facilitated the monomer adsorption effect. To infer this phenomenon, the authors used an excess amount of hardener (*r* = 1.2) and determined the reaction enthalpy by dynamic DSC and obtained higher values (109.0, 98.3, and 105.2, resp., for mixtures containing 0, 0.1, and 0.25 wt% of CNTs) compared to systems with stoichiometric ratio (92.3, 80.8, and 88.8, resp., for mixtures containing 0, 0.1, and 0.25 wt% of CNTs) which implies different reactivity of the primary and secondary amines. Molecular simulation confirmed the consequence of DDM adsorption into CNTs and reasoned the lower values of cure enthalpy after amine functionalization. The shift of peak temperature only observed for systems filled with 0.1 wt% which can be attributed to the constrained mobility of epoxy chains as a consequence of anchored amine groups on the surface of CNTs. Applying *Kamal* equation on data obtained by isothermal calorimetry demonstrated that the noncatalytic constant in this kinetic model does not vary, neither with CNTs addition nor stoichiometry ratio. On the other hand, the autocatalytic constant significantly decreases for epoxy nanocomposites. The retardation effect of carbon nanotubes was markedly lower when the content of CNT was 0.1 wt% due to depression in the extent of autocatalytic mechanism. Besides, the *T*_*g*_ of the epoxy matrix increased by introducing CNTs, because of constrained mobility. This hindrance was more pronounced when an excess amine was used (*r* = 1.2). It is to be noted that the cure behavior of epoxy composites containing functionalized CNTs is challengeable due to variety and/or complexity of reaction kinetics which can be influenced by several dependent variables.  Table 1 represents the above-mentioned works on dealing with cure kinetics of functionalized MWCNTs/epoxy nanocomposites.

## 5. Conclusion

The effect of functionalization of multi-walled carbon nanotubes (MWCNTs) on cure kinetics of epoxy composites is comprehensively discussed based on the available literature. Among various types of surface treatments, the carboxylation and amidation methods were particularly surveyed because of their importance as well as well-documented data. The extent of retardation and/or acceleration in curing, when utilizing untreated and functionalized MWCNTs, was also considered in regard to isothermal or nonisothermal differential scanning calorimetry (DSC). On the basis of dynamic DSC thermograms it can be realized that the functionalization of MWCNTs significantly facilitates the early stage of curing; even though, the cure mechanism remains almost unaffected by the surface modification of the nanotubes. In a different way, the isothermal DSC measurement implies that the incorporation of functionalized MWCNTs has minor effect on vitrification, compared with untreated nanotubes, as a consequence of constrained mobility of polymer chains which arises mainly from intensified interactions between the host polymer and nanotubes. In other words, the state of dispersion is greatly sensitive to either the presence or the type of functional groups used. Employing the phenomenological model proposed by *Kamal* it can be realized that functionalization of MWCNTs slightly improves the rate of autocatalytic reaction. By contrast, the extent of autocatalytic reaction is greatly governed by several interactive factors, for example, curing temperature, stoichiometric ratio of amine/epoxide, and the amount of modified MWCNTs, regardless of the kind of ligands on the surface of CNTs.

## Figures and Tables

**Table 1:** The effect of functionalization of MWCNTs on cure kinetics of epoxy systems.

CNT type	Modification method	Surface modifier	Epoxy type	Curing condition	Reference
MWCTNs	Carboxylation	–COOH	Liquid-crystalline epoxy: (4,40-dihydroxy-and -methyl-stilbene; sulfanilamide)	Isothermal	Bae et al. [12]
MWCNTs	Carboxylation	COOH–	DGEBA: EMI-2,4	Nonisothermal	Zhou et al. [21]
MWCNTs	Carboxylation and fluorination	–COOH and –F	DGEBA: EPIKURE	Isothermal	Abdalla et al. [15], Kim et al. [26]
MWCNTs	Amino-functionalization	–NH	Epoxy resin E51: aromatic curing agent (JX-011)	Nonisothermal	Shen et al. [53, 65]
MWCNTs	Amino-functionalization	–NH	EPON828: EMI-2,4	Nonisothermal	Domínguez et al. [9], Yang et al. [10]
MWNTs	Amino-functionalization	–NH	DGEBA: 4,4′-methylene dianiline	Isothermal and nonisothermal	Choi et al. [16]
MWCNTs	Amino-functionalization	–NH	DGEBA: DDM	Nonisothermal	Prolongo et al. [14]
